# Considerations on male infertility in genital infections with Chlamydia Trachomatis (CT)

**Published:** 2013-09-25

**Authors:** A Al-Moushaly

**Affiliations:** Ghencea Medical Center, Bucharest, Romania; University Emergency Hospital, Bucharest, Romania

**Keywords:** infertility, spermogram, oligozoospermia, asthenozoospermia, teratozoospermia

## Abstract

**Aim: **The study tries to highlight the implication of CT infection in male infertility. **Material and methods: **There were 857 male patients enrolled in the study. The male cases have been investigated in the Gynecology and Urology Department of Ghencea Medical Center, Bucharest. All the selected cases have been done a spermogram test and for the diagnosis of the Chlamydia Trachomatis infection, the ELISA test was used. The test detects the presence in the serum of the anti-chlamydia specific antibodies of type IgA, IgM, IgG (BAG-Chlamydia-AIA). **Results and discussions: **From the total of the investigated subjects, only 233 cases had a modified spermogram. According to the specialty literature, the human factor is involved in 35% of the infertility cases, the male one in 30%, in 20% both factors are involved and in 15% of the cases, there is no incriminating cause after complete investigations. Accordingly, the study supports the literature data.

## Introduction

The Chlamydia Trachomatis infections are prevalent all over the world, but many research studies, as well as the screening, and the treatment concentrate on females. The prevalence of Chlamydia infection is similar in men and women. CT is an important pathogen agent responsible for the appearance of urethritis, epididymitis and orchitis in males. The CT role in the development of prostatitis is controversial, but suggests that CT is an etiologic agent with an incidence of up to 39,5% reported in patients suffering from prostatitis [**12**]. The testicular and prostate infections determine the deterioration of the sperm quality, the fertility being this way affected. Moreover, the Chlamydia infection can affect male fertility by a direct deterioration of the sperm quality: the parameters of the sperm, the DNA fragmentation percent and the acrosome reaction capacity being impaired [**1,2**]. 

Moreover, the proportion of male partners in the infertile couples, who suffer from a Chlamydia infection, is higher than the one registered in the general population. 

 Chlamydia Trachomatis is the most frequent cause of non-gonococcal urethritis and post-gonococcal urethritis in males after the penicillin treatment [**10,11**]. The most frequent symptoms are the following: dysuria (which is less intense than in gonorrhea) which is rarely accompanied by a urethral leakage. In young men, below 35 years old, Chlamydia Trachomatis is the most frequent cause of epididymitis (inflammation of the epididymis). It is clinically manifested by congestion and scrotum volume enlargement, usually on only one side, accompanied by local pain, urethral leakage and dysuria. Moreover, possible infections of the prostatis and the rectum can occur [**5,6**]. The transmission of these infections is favored by having unprotected sexual relationships, by the existence of multiple sex partners and sexual promiscuity [**3,4,13**]. 

 The importance of this pathogen agent in the infections of the male genital tract is often underestimated. 

 In the current study, we would like to briefly examine the role of the Chlamydia infection in men, and, mostly, its role in the male infertility. The incidence rates reported in the Chlamydia genital infections are underestimated due to the clinical aspect, which is, most of the times, asymptomatic.

## Aim

 The study tries to highlight the implication of CT infection in male infertility. 

## Material and Method

 There were 857 male patients enrolled in the study. The male cases have been investigated in the Gynecology and Urology Department of Ghencea Medical Center, Bucharest. 

 All the selected cases have been done a spermogram test and for the diagnosis of the Chlamydia Trachomatis infection, the ELISA test was used. The test detects the presence in the serum of the anti-chlamydia specific antibodies of type IgA, IgM, IgG (BAG-Chlamydia-AIA).

## Results and discussions

From the total of the investigated subjects, only 233 cases had a modified spermogram. According to the specialty literature, the human factor is involved in 35% of the infertility cases, the male one in 30%, in 20% both factors are involved and in 15% of the cases, there is no incriminating cause after complete investigations. Accordingly, the study supports the literature data. 

The distribution of the subjects with modified spermogram on age groups can be seen in the following figure. 

**Table 1:** Distribution of the subjects with modified spermogram on age groups

Age groups	Number of patients
16 – 19 years old	12
20 – 24 years old	21
25 – 29 years old	63
30 – 34 years old	59
35 – 39 years old	34
40 – 44 years old	29
45 – 49 years old	15

**Fig. 1:**
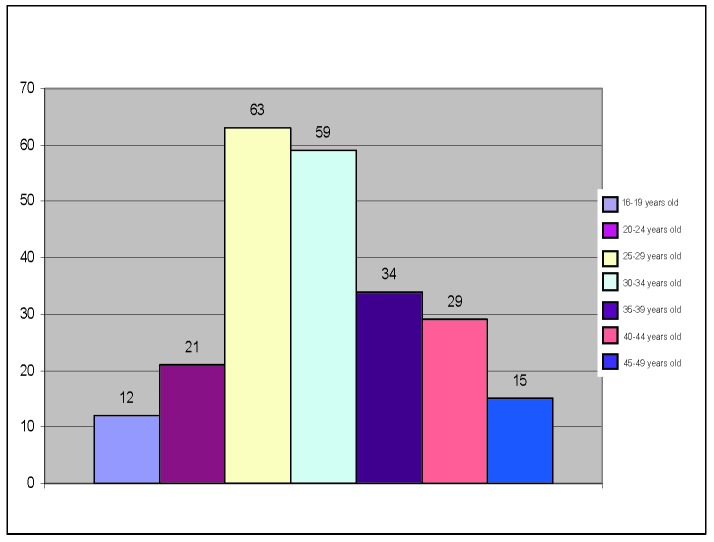
Patients’ distribution according to age groups

The pathology, which is frequently met, responsible for male infertility, was the following: 

 - Oligozoospermia (the concentration of sperm < 20 x 106/ml) 98 cases 

 - Asthenozoospermia (< 50% spermatozoa with progressive movements (A and B category) or < 25% spermatozoa with type A motility): 31 cases 

 - Teratozoospermia (< 30% spermatozoa with normal morphology): 32 cases 

 - Aspermia (the absence of ejaculation): 16 cases 

 - Azoospermia (the absence of spermatozoa in the ejaculation): 56 cases 

**Fig. 2:**
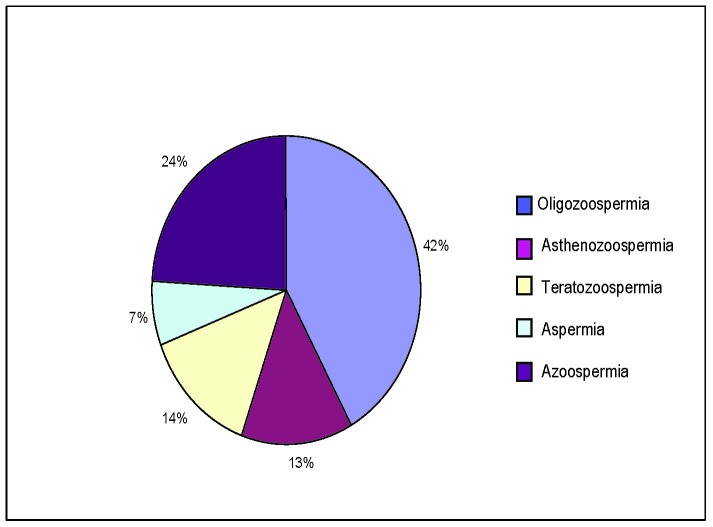
The most frequently met pathology

Oligospermia, which is met in 42% of the cases, bears the following distribution on age groups: 

**Fig. 3:**
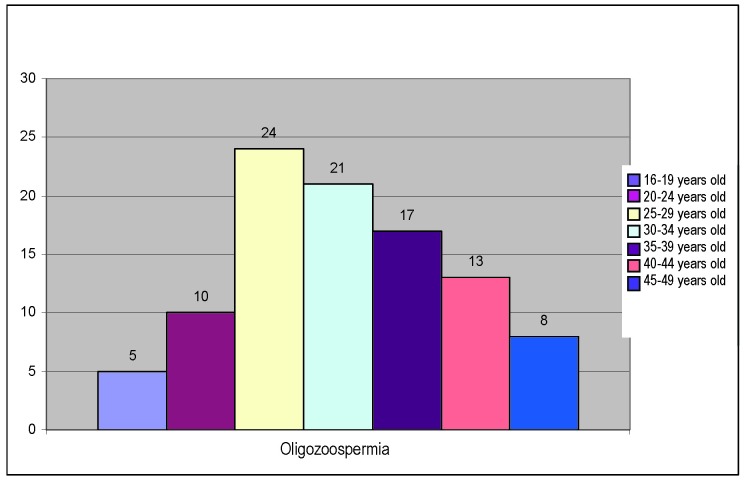
Distribution of oligospermia on age groups

Asthenozoospermia, met in 13% of the cases, is met in the following distribution: 

**Fig. 4:**
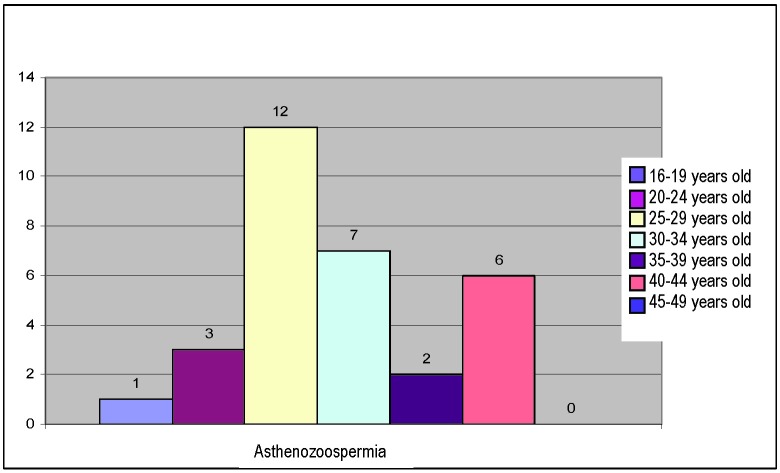
Distribution of asthenozoospermia on age groups

Teratozoospermia, present in 14% of the patients in the studied lot, presents the following age group distribution:

**Fig. 5:**
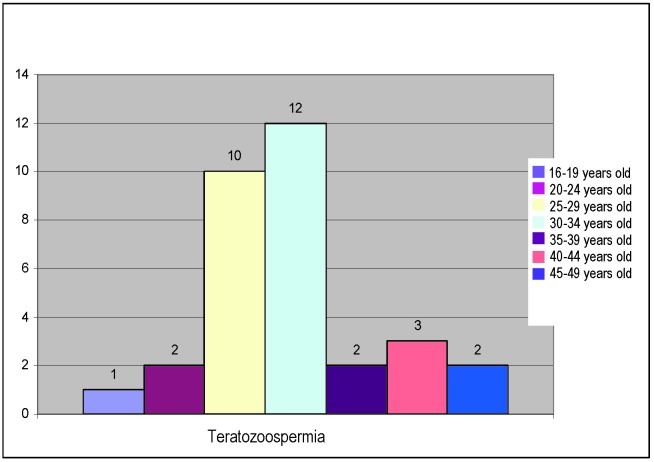
Distribution of teratozoospermia on age groups

16 patients of the studied lot (7%) presented aspermia; the distribution on age groups is presented below. 

**Fig. 6:**
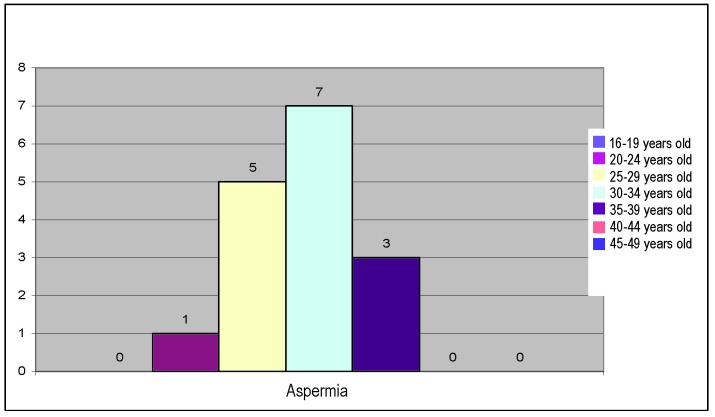
Distribution of aspermia on age groups

Azoospermia, present in 24% of the cases had the following distribution:

**Fig. 7:**
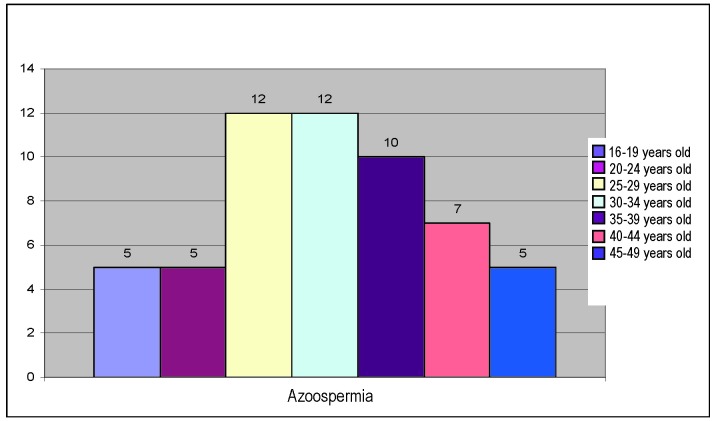
Distribution of azoospermia on age groups

The presence of CT infection during the detection through the serological method has been noticed in 5% (12 cases), out of the 233 cases of modified spermogram.

**Fig. 8:**
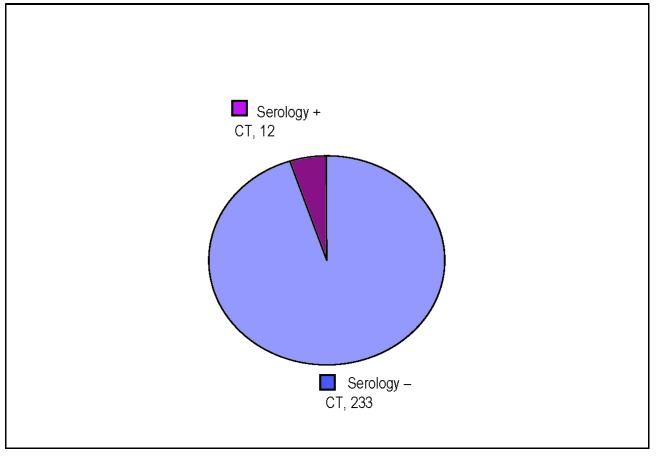
The prevalence of the CT infection in the cases with modified spermogram

**Table 2:** Distribution of the subjects according to age groups, oligozoospermia, asthenozoospermia, teratozoospermia, aspermia, azoospermia

	Oligozoospermia	Asthenozoospermia	Teratozoospermia	Aspermia	Azoospermia
16 - 19 years old	5	1	1	0	5
20 - 24 years old	10	3	2	1	5
25 - 29 years old	24	12	10	5	12
30 - 34 years old	21	7	12	7	12
35 - 39 years old	17	2	2	3	10
40 - 44 years old	13	6	3	0	7
45 - 49 years old	8	0	2	0	5
Total	98	31	32	16	56

The presence of CT infection as a result of the detection by the serological method has been noticed in 5% (12 cases) of 233 cases with modified spermogram. 

Numerous studies in the specialty literature demonstrate the interaction of CT with the spermatozoa. In the electronic microscopy, the testicular biopsy samples and at the level of the epididymis, this interaction of CT with D, H and I stereotypes can be observed, being attached to the in vitro human spermatozoa and the attachment reaction of CT in the sperm from the peritoneal liquid in women with salpingitis could also be noticed [**7,8,11**].

The conclusive proof for the direct effect of Chlamydia on spermatozoa is evidenced by the effect of tyrosine phosphorylase on the proteins in the sperm. 

Moreover, the anti CT antibodies, serotype E and LGV can lead to the apoptosis of the spermatozoon in vitro. 

A study was done on 627 cases, of which 136 presented a CT infection. In these patients, it could be observed that the presence of Chlamydia affects the morphology of the spermatozoa in 14,4% of the cases, volume to 6,4%, concentration to 8,3%, mobility to 7,8% and speed to 9,3%. 

Chlamydia Trachomatis and male infertility 

In the specialty literature, it is known that the CT infection produces a series of urethritis as well as infections of the accessory sexual glands; however, the CT role as an etiological agent of male infertility and the presence of CT in the sperm, represent a very controversial problem at present [**9,10**]. In addition, no (inter-) relationships between the IgG class CT antibodies have been detected as a marker of some previous infections and the quality of the sperm. However, the IgG (anti-CT) presence cannot represent an accurate indicator of the previous exposures due to the testicular barrier, but the presence of the anti-CT antibodies in the sperm was mostly correlated with the partner’s infertility. 

## Conclusions 

• The Chlamydia Trachomatis infection leads to urethritis and infections of the accessory sexual glands. However, the CT role, as an ethological agent of male infertility, and the presence of CT in the sperm is very controversial at present. 

 • An amount of 233 cases out of the total 865 cases found through screening methods, have had a modified spermogram: 98 cases of oligospermia, 31 cases of asthenozoospermia, 32 cases of teratozoospermia, 16 cases of aspermia and 56 cases of azoospermia. 

 • The presence of the CT infection, as a result of the detection by serological method, has been shown in 5% (12 cases) of 233 cases of modified spermogram. 

 • In conclusion, it can be said that the infertility produced by the CT infection seems to be a consequence of sexual transmission, leading to the appearance of a tubal disease with a consecutive sterility of the partner than by directly influencing the male reproductive function.

## References

[1] Sahleanu  V, Maicanescu  GM (1996). Introducere in sexologie masculine.

[2] Guaschino  S, De Seta  F (2000). Update on Chlamydia trachomatis. Annals of the New York Academy of Sciences.

[3] Ness  RB, Markovic  N (1997). Do men become infertile after having sexually transmitted urethritis? An epidemiologic examination. Fertil Steril.

[4] Mutlu  N, Mutlu  B (1998). The role of Chlamydia trachomatis in patients with non-bacterial prostatitis. Int J Clin Pract.

[5] Centers for Disease Control and Prevention Fact sheet for public health personnel: male latex condoms and sexually transmitted diseases. Centers for Disease Control and Prevention, National Center for HIV, STD and TB Prevention, 2004..

[6] Centers for Disease Control and Prevention, U.S. Department of Health and Human Services (2010). Sexually Transmitted Diseases Treatment Guidelines.

[7] Chen  MY, Rohrsheim  R (2007). The differing profiles of symptomatic and asymptomatic Chlamydia trachomatis-infected men in a clinical setting. Int J STD AIDS.

[8] Shafer  MA, Schachter  J (1993). Evaluation of urine-based screening strategies to detect Chlamydia trachomatis among sexually active asymptomatic young males. JAMA.

[9] Hosseinzadeh  S, Brewis  IA (2001). Co-incubation of human spermatozoa with Chlamydia trachomatis serovar E causes premature sperm death. Hum Reprod.

[10] Eley  A, Hosseinzadeh  S (2005). Apoptosis of ejaculated human sperm is induced by coincubation with Chlamydia trachomatis lipopolysaccharide. Hum Reprod.

[11] Ginocchio  RH, Veenstra  DL (2003). The clinical and economic consequences of screening young men for genital chlamydial infection. Sex Transm Dis.

[12] Shafer  MA, Schachter  J (1993). Evaluation of urine-based screening strategies to detect Chlamydia trachomatis among sexually active asymptomatic young males. JAMA.

[13] Ostaszewska-Puchalska I, Zdrodowska-Stefanow  B (2004). Antichlamydial antibodies in the serum and expressed prostatic secretion in prostatitis. Arch Immunol Ther Exp (Warsz).

